# Effects of altitude on the gut microbiome and metabolomics of Sanhe heifers

**DOI:** 10.3389/fmicb.2023.1076011

**Published:** 2023-02-24

**Authors:** Xinyu Zhang, Wei Wang, Zhijun Cao, Hongjian Yang, Yajing Wang, Shengli Li

**Affiliations:** State Key Laboratory of Animal Nutrition, College of Animal Science and Technology, China Agricultural University, Beijing, China

**Keywords:** different altitudes, gut microbiota, metabolites, Sanhe heifers, high-altitude adaptation

## Abstract

**Introduction:**

Extreme environments at high altitudes pose a significant physiological challenge to animals. We evaluated the gut microbiome and fecal metabolism in Sanhe heifers from different altitudes.

**Methods:**

Twenty Sanhe heifers (body weight: 334.82 ± 13.22 kg, 15-month-old) selected from two regions of China: the Xiertala Cattle Breeding Farm in Hulunbeier, Inner Mongolia [119°57′ E, 47°17′ N; approximately 700 m altitude, low altitude (LA)] and Zhizhao Dairy Cow Farm in Lhasa, Tibet [91°06′ E, 29°36′ N; approximately 3,650 m altitude, high altitude (HA)], were used in this study. Fecal samples were collected and differences in the gut microbiota and metabolomics of Sanhe heifers were determined using 16S rRNA gene sequencing and metabolome analysis.

**Results and discussion:**

The results showed that altitude did not significantly affect the concentrations of fecal volatile fatty acids, including acetate, propionate, butyrate, and total volatile fatty acids (*p* > 0.05). However, 16S rRNA gene sequencing showed that altitude significantly affected gut microbial composition. Principal coordinate analysis based on Bray–Curtis dissimilarity analysis revealed a significant difference between the two groups (*p* = 0.001). At the family level, the relative abundances of Peptostreptococcaceae, Christensenellaceae, Erysipelotrichaceae, and Family_XIII were significantly lower (*p* < 0.05) in LA heifers than in HA heifers. In addition, the relative abundances of Lachnospiraceae, *Domibacillus*, Bacteroidales_S24-7_group, Bacteroidales_RF16_group, Porphyromonadaceae, and Spirochaetaceae were significantly higher in HA heifers than in LA heifers (*p* < 0.05). Metabolomic analysis revealed the enrichment of 10 metabolic pathways, including organismal systems, metabolism, environmental information processing, genetic information processing, and disease induction. The genera *Romboutsia*, *Paeniclostridium*, and *g_unclassified_f_Lachnospiraceae* were strongly associated with the 28 differential metabolites. This study is the first to analyze the differences in the gut microbiome and metabolome of Sanhe heifers reared at different altitudes and provides insights into the adaptation mechanism of Sanhe heifers to high-altitude areas.

## 1. Introduction

Areas with an altitude higher than 2,500 m, generally defined as high altitudes (Moore et al., 2010), pose challenges for the survival, growth, and development of local animals (Moore et al., 2010; Guo et al., 2014). As the highest plateau worldwide, the Tibetan Plateau is characterized by low pressure, low oxygen, strong ultraviolet rays, and low temperatures throughout the year (Zha et al., 2016; Sun et al., 2021). High altitude, low pressure, and hypoxia can cause various diseases such as pulmonary hypertension (Pasha and Newman, 2010; Khanna et al., 2018), high-altitude hypertension (Bilo et al., 2019), and vascular dysfunction. Additionally, numerous metabolic disorders in the digestive tract, such as enteritis and gastritis, can be caused by an imbalance in the gut microbiota under low pressure and hypoxic conditions (Khanna et al., 2018; Hill et al., 2020; Pena et al., 2022).

Bacteria play a key role in many types of feed biopolymer fermentation and degradation processes (Bickhart and Weimer, 2018), and fecal samples mostly represent the distal portion of the gut microbiotathe (de Oliveira et al., 2013). Recent studies have reported the high-altitude adaptability of various animals such as cattle (Kong et al., 2021), yak (Ayalew et al., 2021), and rats (Murray, 2016). Recently, alterations in the gastrointestinal microbiota due to altitude changes were investigated in yak (Liu et al., 2021), sheep (Holman et al., 2019), and pigs (Zeng et al., 2020). However, the changes in the gut microbiota and the physiological and metabolic mechanisms in response to high altitudes have not yet been investigated in Sanhe heifers. Moreover, variations in the metabolic adaptability of the gut microbiota of Sanhe heifers at different altitudes are not well understood.

As a dual-purpose breed, Sanhe cattle show excellent performance in both milk and meat production (Xu et al., 2017); their milk contains high concentrations of fat. In addition, Sanhe cattle exhibit strong adaptability, rough feeding tolerance (Hu et al., 2019), strong disease resistance (Usman et al., 2017), and stable genetic performance. Sanhe cattle originated from Inner Mongolia, China, and were bred to result in multiple breeds, including native Mongolian cattle, Simmental cattle, Siberian cattle, improved Russian cattle, Zabaikal cattle, Tagil cattle, Yaroslav cattle, Swedish cattle, and Hokkaido Dutch cattle. We examined whether Sanhe cattle can adapt to high-altitude environments, by evaluating the differences between Sanhe heifers in low-and high-altitude regions from a gut microbiota perspective.

In this study, the gut microbiomes of Sanhe heifers being offered the same amount of nutrients by the total mixed ration (TMR) but living in two regions of different altitudes, were compared. Using high-throughput sequencing and LS-MS-based untargeted metabolome analyzes, we aimed to reveal the effect of altitude on the gut microbiome of Sanhe cattle and to improve the understanding of the role of the gut microbiome in high-altitude adaptability. In addition, the gut microbiome and metabolomics of Sanhe heifers from low-and high-altitude regions were compared to provide insights into the high-altitude adaptability of ruminants.

## 2. Materials and methods

The study protocol was approved by the Ethical Committee of the College of Animal Science and Technology of China Agricultural University (project number AW22121202-1-2).

### 2.1. Experimental animals

Twenty Sanhe heifers (body weight: 334.82 ± 13.22 kg, 15-month-old) fed in two different altitude regions of China were selected for the experiments. Ten Sanhe heifers were selected from each trial site.

### 2.2. Study regions and management

Cattle from two regions were analyzed: those from the origin of Sanhe cattle, including Hulunbuir, Inner Mongolia Autonomous Region (119°57′ E, 47°17′ N; approximately 700 m in altitude, LA group), and Lhasa, Tibet Autonomous Region (91°06′ E, 29°36′ N; approximately 3,650 m in altitude, HA group). Lhasa has an average annual temperature of 8.6 Â °C and annual precipitation of 472.5 mm (Fan et al., 2005), whereas Hulunbuir has an average annual temperature of 3.3°C and annual precipitation of 538.3 mm.

### 2.3. Fecal sample collection and processing

To obtain representative samples, feces from Sanhe heifers were collected from the rectum using plastic gloves. The fecal samples used to analyze the gut microbiota were immediately frozen in liquid nitrogen (−80°C), and those used to analyze volatile fatty acids (VFAs) were stored at −20°C. For VFA analysis, the fecal sample from each animal was thawed, diluted, and centrifuged at 8,000 × *g* at 4°C for 10 min, and the supernatant was collected and evaluated using gas chromatography (Erwin et al., 1961).

### 2.4. Genomic DNA extraction, PCR amplification, and 16S rRNA sequencing

Total microbial genomic DNA was extracted from 1 g of fecal samples using an OMEGA kit (Omega Bio-Tek, Norcross, GA, United States), following the manufacturer’s instructions. A Nanodrop 2000 spectrophotometer (Thermo Fisher Scientific, Waltham, United States) was used to confirm the purity and concentration of the extracted DNA. The V3–V4 region of the gut bacterial 16S rRNA gene was amplified using the forward primer 338F (5′-ACTCCTACGGGAGGCAGCA-3′) and the reverse primer 806R (3′-GGACTACNNGGGTATCTAAT-5′). The PCR conditions were as follows: denaturation at 95°C for 5 min, followed by 28 cycles at 95°C for 45 s, 55°C for 50 s, and 72°C for 45 s, with a final extension at 72°C for 10 min. Amplified fragments were visualized using 2% agarose gel electrophoresis, and the respective bands were purified using an Agencourt AMPure XP kit (Beckman Coulter Genomics, Brea, CA, United States) according to the manufacturer’s instructions and quantified using QuantiFluor-ST (Promega, Madison, WI, USA). Purified PCR products were sequenced on an Illumina MiSeq (Illumina, San Diego, CA, United States; Caporaso et al., 2012) using a 2 × 250 bp sequencing kit.

### 2.5. Quality control and statistical analysis

Sequences with scores ≤20 (low quality), reads <200 bp, and reads containing ambiguous bases or unmatched primer sequences were filtered out using QIIME 1.8 (Caporaso et al., 2010), and barcode tags were removed. The obtained sequences were combined using PEAR 0.9.6 (Zhang et al., 2014) and demultiplexed using Flash (version 1.20; Mago and Salzberg, 2011). Reads with a combined length of <230 bp and chimeric sequences were removed using the UCHIME algorithm (Edgar et al., 2011). To reduce errors due to different sequencing depths, all samples were subsampled to an equal size of 31,719 for downstream alpha-and beta-diversity analyzes. To ensure comparability of species diversity between samples, standardized operational taxonomic unit (OTU) documents were used to analyze the species and diversity indices.

The resulting sequences were clustered into OTUs based on a 97% sequence similarity threshold using the Ribosomal Database Project classifier (Cole et al., 2009) with a confidence threshold of 0.70 and compared against the SILVA 128 database for microbial species annotation (Quast et al., 2012). All OTUs were removed using UCLUST to generate the representative OTU table (Edgar, 2010).

The OTU level alpha diversity of the bacterial communities was determined using the Chao1, Shannon, and Simpson indices and procedures within QIIME 1.8, and visualized using the “ggplot2” package of R (version 4.0.5; Wickham, 2009). Principal coordinate analysis (PCoA) based on the Bray–Curtis dissimilarity matrix was performed in R using the “vegan” package for beta diversity analysis (Oksanen et al., 2016).

### 2.6. Metabolite extraction

The cold extraction solvent methanol/acetonitrile/H_2_O (2:2:1, vol/vol/vol; 1 ml) was added to an 80 mg fecal sample and vortexed for 60 s to extract metabolites. The samples were incubated on ice for 20 min and centrifuged at 14,000 × *g* for 20 min at 4°C. The supernatant was collected for liquid chromatography (LC)-MS analysis. The samples were dissolved in 100 μl of acetonitrile/water (1:1, v/v) and transferred to LC vials.

### 2.7. Liquid chromatography–MS/MS analysis and data processing

Gut microbiota metabolites were separated using an ultra-high-performance liquid chromatography system (1,290 Infinity LC, Agilent Technologies, Santa Clara, CA, United States) coupled to a quadrupole time-of-flight (TripleTOF 6,600, AB Sciex, Framingham, MA, United States). The fecal samples were analyzed using a 2.1 mm × 100 mm ACQUIY UPLC BEH 1.7 μm column (Waters, Milford, MA, United States). In both the positive and negative electrospray ionization modes, the mobile phase contained 25 mM ammonium acetate and 25 mM ammonium hydroxide in water and acetonitrile, respectively. The gradient was 85% acetonitrile for 1 min, which was linearly reduced to 65% in 11 min, reduced to 40% in 0.1 min and maintained for 4 min, and increased to 85% in 0.1 min, with a 5 min re-equilibration period. The electrospray ionization source conditions were as follows: ion source Gas1 as 60, ion source Gas2 as 60, curtain gas as 30, source temperature, 600°C; and ion spray voltage floating ±5,500 V. During MS acquisition, the instrument was set to acquire signals over an m/z range of 60–1,000 Da, and the accumulation time for the time-of-flight MS scan was set to 0.20 s/spectra. In the auto-MS/MS acquisition mode, the instrument was set to acquire signals over an m/z range of 25–1,000 Da, and the accumulation time for the production scan was set to 0.05 s/spectra. The production scan was acquired using information-dependent acquisition in high-sensitivity mode. The collision energy was fixed at 35 ± 15 eV. The declustering potential was set at ±60 V.

Raw MS data (Wiff. scan files) were converted to MzXML files using ProteoWizard MSConvert, and processed using XCMS for feature detection, retention time correction, and alignment. The metabolites were identified using accuracy mass spectrometry (<25 ppm) and MS/MS data, which were matched with the standard database.

For the extracted ion features, only variables with >50% of the nonzero measurement values in at least one group were retained. The MetaboAnalyst^1^ web-based system was used for multivariate statistical analysis. After Pareto scaling, PCoA and partial least squares discriminant analysis (OPLS-DA) were performed. Leave-one-out cross-validation and response permutation testing were conducted to evaluate the robustness of the model. Metabolites showing significant differences between the LA and HA groups were identified based on the combination of a statistically significant threshold of variable influence on projection (VIP) values obtained from the OPLS-DA model and a two-tailed Student’s *t*-test (*p-*value) on the raw data. The metabolites were considered significant when they had VIP values >1.0, VIP values <0.05, and *p-*values less than 0.05. Differential metabolites were identified using three databases, including the Kyoto Encyclopedia of Genes and Genomes (KEGG)^2^, the human metabolome database, and the bovine metabolome database. The KEGG database was used to evaluate the enrichment analysis of KEGG metabolic pathways according to the differential metabolites (Kanehisa et al., 2012). Fisher’s exact test was used to determine the significance of enriched pathways.

### 2.8. Statistical analysis

Fecal fermentation parameters were analyzed using the *t*-test in the SPSS software (version 22.0, SPSS, Inc., Chicago, IL, United States). Alpha diversity indices, which reflect the significance between the LA and HA groups, were analyzed using the Wilcoxon rank test with the “dplyr” package (authors, H. Wickham, R. François, L. Henry, K. Müller; published date, 2018; version, 0.7.6) in R. PCoA was performed based on the Bray–Curtis dissimilarity matrices in R, and “ggplot2” package in R was used to visualize the results. The differences in the relative abundance of organisms at the phylum, family, and genus levels and microbiota function between the two groups were tested using the Wilcoxon method in R (version 4.0.5). Spearman’s rank correlation was used to identify the relationship between the relative abundance of the core OTUs, altitude, fecal fermentation parameters, and serum antioxidant indices using the “Psych” package (author, W. Revelle; published date, 2016; version, 1.6.9) and visualized using the “corrplot” package (author, Taiyun Wei; published date, 2017; version, 0.84) in R. All data were reported as the mean, and differences with *p* < 0.05 were considered as significant.

## 3. Results

### 3.1. Gut fermentation parameters of Sanhe heifers from different altitudes

As shown in Table 1, there was no significant difference (*p* > 0.05) in the concentrations of acetate, propionate, butyrate, and total VFAs between fecal samples of Sanhe cattle reared at different altitudes. Compared with the LA group, the acetate-to-propionate ratio (A/P) increased significantly (*p* < 0.05) in the HA group.

**Table 1:** Fecal-sample fermentation parameters at Sanhe heifers from different altitudes.

Items	Groups^1^		SEM	*p*-value
	LA	HA		
Acetate, mmol/L	7.412	6.88	0.67	0.40
Propionate, mmol/L	3.92	3.22	0.43	0.06
Butyrate, mmol/L	1.48	1.48	0.20	0.88
AP^2^	1.77^b^	2.13^a^	0.07	0.002
TVFAs^3^, mmol/L	12.40	11.60	1.33	0.52

^1^LA represents the low-altitude region (Hulunbuir, Inner Mongolia Autonomous Region, 119°57′ E, 47°17′ N; approximately 700 m altitude, LA); HA represents the high-altitude region (Lhasa, Tibet Autonomous Region 91°06′ E, 29°36′ N; approximately 3,750 m altitude, HA). ^2^AP, the acetate-to-propionate raito (AP). ^3^TVFA, total volatile fatty acids.The differences among the six groups are indicated by various letters (*p* < 0.05).

### 3.2. Gut microbiota communities of Sanhe heifers from different altitudes

#### 3.2.1. Sequencing metrics for the gut microbiota of heifers

A total of 868,445 raw sequences were generated with an average of 43,422 ± 4,392.71 (mean ± SD) per sample. An average of 2,054 ± 133.75 OTUs across all samples were identified at 3% sequence dissimilarity. Rarefaction curves showed that the number of new OTUs decreased as the number of sequences per sample increased (Additional file: Supplementary Figure S1), indicating an adequate sampling depth to cover the tested gut bacterial composition. Good’s coverage for the Sanhe heifer samples showed a mean value of 0.97 across all 20 samples, indicating sufficient sequence coverage for all samples. The mean Shannon’s diversity and Chao1’s richness for all Sanhe heifer samples were 8.42 ± 0.49 and 2,674.69 ± 157.41 (Additional file: Supplementary Table S2), respectively.

**Figure 1:**
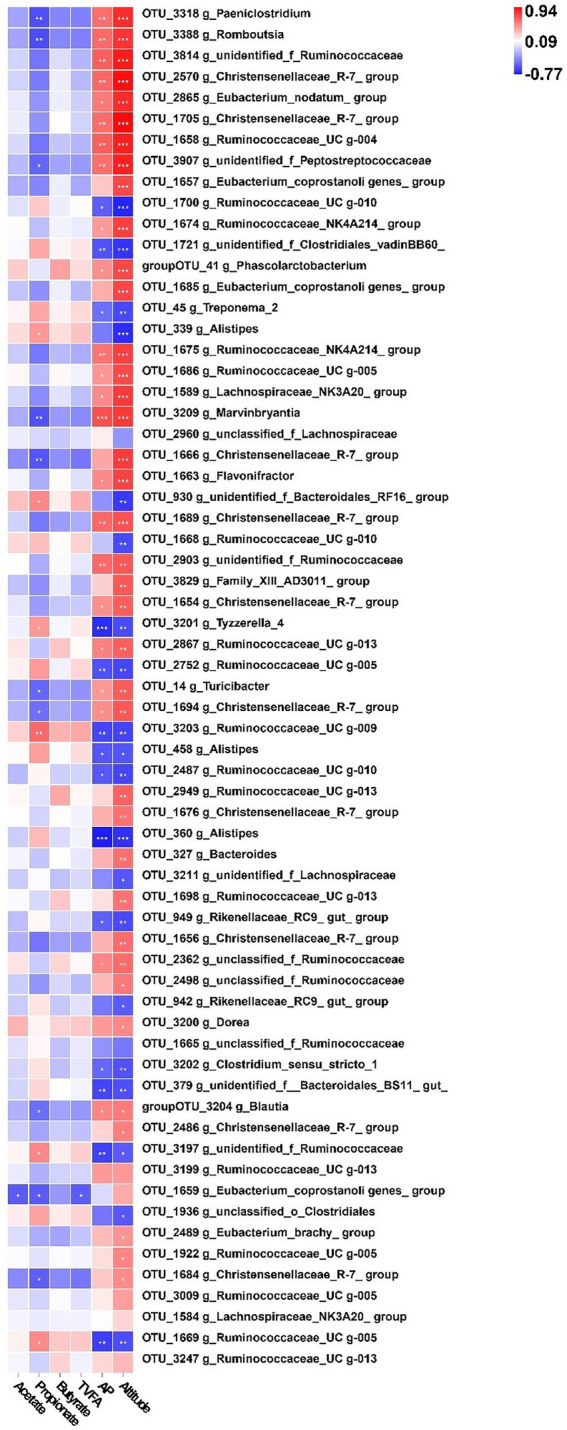
The gut bacterial composition of Sanhe heifers. **(A)** Relative distribution of the most dominant bacterial phyla, family, and genera (relative abundance >0.5% of all the 20 samples) in the low-altitude (LA) and high-altitude (HA) group samples. The inner ring in the pie chart represents the genus level, the middle ring represents the family level, and the outer ring represents the phylum level. Different color shades represent different bacteria. The numbers in brackets denote the average relative abundance of the bacteria across 20 samples. **(B)** Stacked bar graphs of the average relative abundances of phyla, family, and genus (top 20 of relative abundances) for Sanhe heifers in the LA and HA groups. **(C)** Flower diagram plot of Sanhe heifer samples. The core community in all Sanhe heifers was defined as operational taxonomic units present in all Sanhe heifers at all sampling times. **(D)** Principal coordinate analysis (PCoA) plots of bacterial communities in Sanhe heifer samples.

The most highly abundant phyla in all Sanhe heifer samples were Firmicutes (64.06%), Bacteroidetes (32.33%), Tenericutes (0.93%; Figures 1A,B). Among these phyla, the most abundant families were Ruminococcaceae (36.37%), Rikenellaceae (15.43%), Peptostreptococcaceae (12.23%), and Christensenellaceae (5.51%; Figures 1A,B). At the genus level, 11 genera showed >2% relative abundance (Figures 1A,B).

#### 3.2.2. Defining the core gut microbiota for Sanhe heifers in this study

The intestinal microbiome of the Sanhe cattle varies widely. Therefore, we focused on the core OTUs found in all the Sanhe heifers. We sought to identify the core microbiota across all Sanhe heifers and found 393 shared OTUs among all samples from LA and HA Sanhe heifers, as shown in Figure 1C. These OTUs included the following bacterial families with *>*10% total relative abundance: Ruminococcaceae (26.10%), Rikenellaceae (11.37%, Figures 1A,B). The shared genera among all samples showing >5% of the total relative abundance were *Ruminococcaceae_UCG-005* (13.71%), *Rikenellaceae_RC9_gut_group* (7.74%; Figures 1A,B).

#### 3.2.3. Differences in the gut bacterial community between LA and HA Sanhe heifers

To detect differences in the gut microbiota of LA and HA Sanhe heifers, we performed Bray–Curtis dissimilarity analysis. The results were visualized using a principal coordinate analysis (PCoA) plot, as shown in Figure 1D. The gut microbiota that differed between groups were analyzed using analysis of similarities and confirmed that the two groups significantly differed (*R*^2^ = 0.58, *p* = 0.001). However, we found no significant difference (*p* < 0.05) in Chao 1 richness, Shannon diversity index, and Simpson’s diversity index between the groups (Additional file: Supplementary Table S2).

At the phylum level, the relative abundances of the phyla Firmicutes, Bacteroidetes, and Verrucomicrobia did not differ significantly (*p* > 0.05) between the LA and HA groups. In contrast, compared with the LA group, the relative abundances of the phyla Proteobacteria and Actinobacteria were significantly (*p* < 0.05) lower (Table 2), whereas that of the phylum Spirochaetae was significantly (*p* < 0.05) higher in the HA group. At the family level (family of relative abundance >0.01%), lower relative abundances of Peptostreptococcaceae, Christensenellaceae, Erysipelotrichaceae, Family_XIII, Acidaminococcaceae, Peptococcaceae, Enterobacteriaceae, Spirochaetaceae and Coriobacteriaceae were observed in the HA group than in the LA group (Table 2), and the relative abundances of Lachnospiraceae, Clostridiales_vadinBB60_group, Bacteroidales_S24-7_group, Bacteroidales_RF16_group, and Porphyromonadaceae were higher in the LA group than in the HA group (Table 2). At the genus level (genera with relative abundance >0.01%), compared with the LA group, the relative abundances of 43 genera were significantly (*p* < 0.05) higher, and those of 15 genera were significantly lower in the HA group (Table 3); of these, eight genera showed a relative abundance >1%. The relative abundances of some genera differed by more than 10-fold, including *Butyrivibrio* (decreasing 20.59-fold, *p* = 0.002), *Eubacterium_xylanophilum_group* (decreasing 13.25-fold, *p* < 0.001), *Corynebacterium* (increasing 16.51-fold, *p* = 0.011), *Escherichia-Shigella* (increasing 213.31-fold, *p* < 0.001), and *Domibacillus* (increasing from 0.00 to 0.027, *p* < 0.001; Table 3).

**Table 2:** Phyla and Families of relative abundance >0.1% within the gut microbiota of LA and HA heifers.

Phyla/families	Groups^1^		SEM	*p*-value
	LA	HA		
Firmicutes	67.078	61.035	1.764	0.105
Ruminococcaceae	35.082	37.653	1.231	0.190
Peptostreptococcaceae	12.234	4.016	1.142	<0.001
Lachnospiraceae	4.796	10.162	1.855	0.043
Christensenellaceae	7.249	3.767	0.568	0.002
Erysipelotrichaceae	2.409	1.244	0.204	0.003
Family_XIII	2.044	1.151	0.142	0.002
Clostridiales_vadinBB60_group	0.223	0.691	0.067	<0.001
Acidaminococcaceae	0.700	0.202	0.079	0.001
Clostridiaceae_1	0.204	0.283	0.024	0.140
Peptococcaceae	0.175	0.104	0.016	0.010
Bacteroidetes	29.247	35.422	1.684	0.089
Rikenellaceae	13.138	17.715	1.129	0.075
Prevotellaceae	5.883	4.364	0.594	0.105
Bacteroidaceae	4.266	3.671	0.325	0.123
Bacteroidales_S24-7_group	0.794	2.163	0.345	0.011
f_p-2,534-18B5_gut_group	1.383	1.394	0.373	0.520
Bacteroidales_Incertae_Sedis	1.166	1.424	0.122	0.529
Bacteroidales_RF16_group	0.470	1.818	0.209	0.001
Porphyromonadaceae	0.343	0.766	0.089	0.005
Bacteroidales_BS11_gut_group	0.265	0.382	0.041	0.290
Bacteroidales_UCG-001	0.194	0.066	0.031	0.064
Proteobacteria	0.505	0.192	0.087	0.016
Enterobacteriaceae	0.332	0.007	0.089	<0.001
Spirochaetae	0.161	1.081	0.152	<0.001
Spirochaetaceae	0.161	1.081	0.152	<0.001
Verrucomicrobia	0.499	0.373	0.066	0.218
Verrucomicrobiaceae	0.432	0.338	0.058	0.436
Actinobacteria	0.870	0.300	0.109	0.001
Coriobacteriaceae	0.514	0.246	0.048	0.001
Bifidobacteriaceae	0.283	0.044	0.097	0.472

^1^LA represents the low-altitude region (Hulunbuir, Inner Mongolia Autonomous Region, 119°57′ E, 47°17′ N; approximately 700 m altitude, LA); HA represents the high-altitude region (Lhasa, Tibet Autonomous Region 91°06′ E, 29°36′ N; approximately 3,750 m altitude, HA).

**Table 3:** Genera of relative abundance >0.01% within the gut microbiota of LA and HA heifers as determined using the Wilcoxon test (only *p* < 0.05 is shown).

Genera	Groups^1^		SEM	*P*-value
	LR	HR		
Christensenellaceae_*R*-7_group	3.707	7.189	0.565	0.002
Ruminococcaceae_UCG-010	6.610	4.059	0.435	<0.001
*Alistipes*	4.902	3.306	0.329	0.023
*Paeniclostridium*	1.689	6.349	0.648	<0.001
*Romboutsia*	1.959	4.773	0.406	<0.001
Ruminococcaceae_UCG-013	2.473	3.885	0.225	0.001
Prevotellaceae_UCG-003	1.718	2.878	0.389	0.029
*Turicibacter*	1.033	2.172	0.204	0.006
Family_XIII_AD3011_group	0.626	1.035	0.091	0.029
Ruminococcaceae_NK4A214_group	0.564	1.054	0.072	<0.001
dgA-11_gut_group	0.502	0.855	0.070	0.015
*Treponema*_2	1.034	0.153	0.145	<0.001
Lachnospiraceae_NK3A20_group	0.402	0.683	0.056	0.011
*Phascolarctobacterium*	0.199	0.696	0.079	<0.001
*Tyzzerella*_4	0.569	0.268	0.058	0.003
*Ruminococcus*_1	0.432	0.243	0.041	0.023
*Flavonifractor*	0.177	0.353	0.027	<0.001
*Eubacterium*_brachy_group	0.188	0.315	0.025	0.013
*Eubacterium*_oxidoreducens_group	0.335	0.118	0.055	0.007
Ruminococcaceae_UCG-004	0.075	0.376	0.040	<0.001
*Candidatus*_Soleaferrea	0.258	0.174	0.016	0.006
Ruminococcaceae_UCG-011	0.086	0.324	0.033	<0.001
*Eubacterium*_*nodatum*_group	0.081	0.307	0.032	<0.001
*Intestinibacter*	0.106	0.256	0.024	0.002
*Butyrivibrio*	0.335	0.016	0.053	0.003
Dorea	0.133	0.212	0.018	0.017
*Escherichia*-*Shigella*	0.007	0.332	0.089	<0.001
*Marvinbryantia*	0.097	0.201	0.016	0.002
*Blautia*	0.098	0.200	0.021	0.021
Lachnospiraceae_AC2044_group	0.154	0.067	0.015	0.009
*Acetitomaculum*	0.025	0.189	0.026	<0.001
Lachnospiraceae_UCG-010	0.074	0.137	0.013	0.028
*Ruminiclostridium*_9	0.058	0.135	0.016	0.004
*Pseudobutyrivibrio*	0.101	0.061	0.010	0.049
*Olsenella*	0.038	0.096	0.009	0.001
*Anaerorhabdus_furcosa*_group	0.044	0.074	0.008	0.015
*Oscillospira*	0.045	0.071	0.007	0.017
*Oscillibacter*	0.033	0.079	0.008	0.002
*Anaerovorax*	0.034	0.072	0.009	0.019
Lachnospiraceae_UCG-001	0.033	0.063	0.006	0.004
*Anaerosporobacter*	0.021	0.072	0.010	0.016
*Eubacterium_hallii*_group	0.028	0.052	0.005	0.012
*Ruminococcus_gauvreauii*_group	0.012	0.049	0.007	0.003
*Gordonibacter*	0.011	0.048	0.009	0.006
Family_XIII_UCG-001	0.017	0.040	0.004	0.007
*Atopobium*	0.012	0.042	0.005	0.002
*Faecalitalea*	0.010	0.041	0.006	0.012
*Senegalimassilia*	0.014	0.037	0.004	0.003
*Elusimicrobium*	0.031	0.009	0.005	0.006
Family_XIII_UCG-002	0.007	0.032	0.004	0.005
Erysipelotrichaceae_UCG-004	0.033	0.006	0.006	0.004
Lachnospiraceae_UCG-002	0.005	0.031	0.004	<0.001
*Corynebacterium*_1	0.004	0.027	0.004	<0.001
*Solobacterium*	0.006	0.022	0.002	<0.001
*Corynebacterium*	0.002	0.026	0.007	0.011
*Domibacillus*	0.000	0.027	0.004	<0.001
Anaeroplasma	0.018	0.007	0.003	0.025
*Eubacterium_xylanophilum*_group	0.021	0.002	0.003	<0.001

^1^LA represents the low-altitude region (Hulunbuir, Inner Mongolia Autonomous Region, 119°57′ E, 47°17′ N; approximately 700 m altitude, LA); HA represents the high-altitude region (Lhasa, Tibet Autonomous Region 91°06′ E, 29°36′ N; approximately 3,750 m altitude, HA).

#### 3.2.4. Correlation of core gut bacteria with altitude, and gut fermentative parameters in HA and LA Sanhe heifers

**Figure 2:**
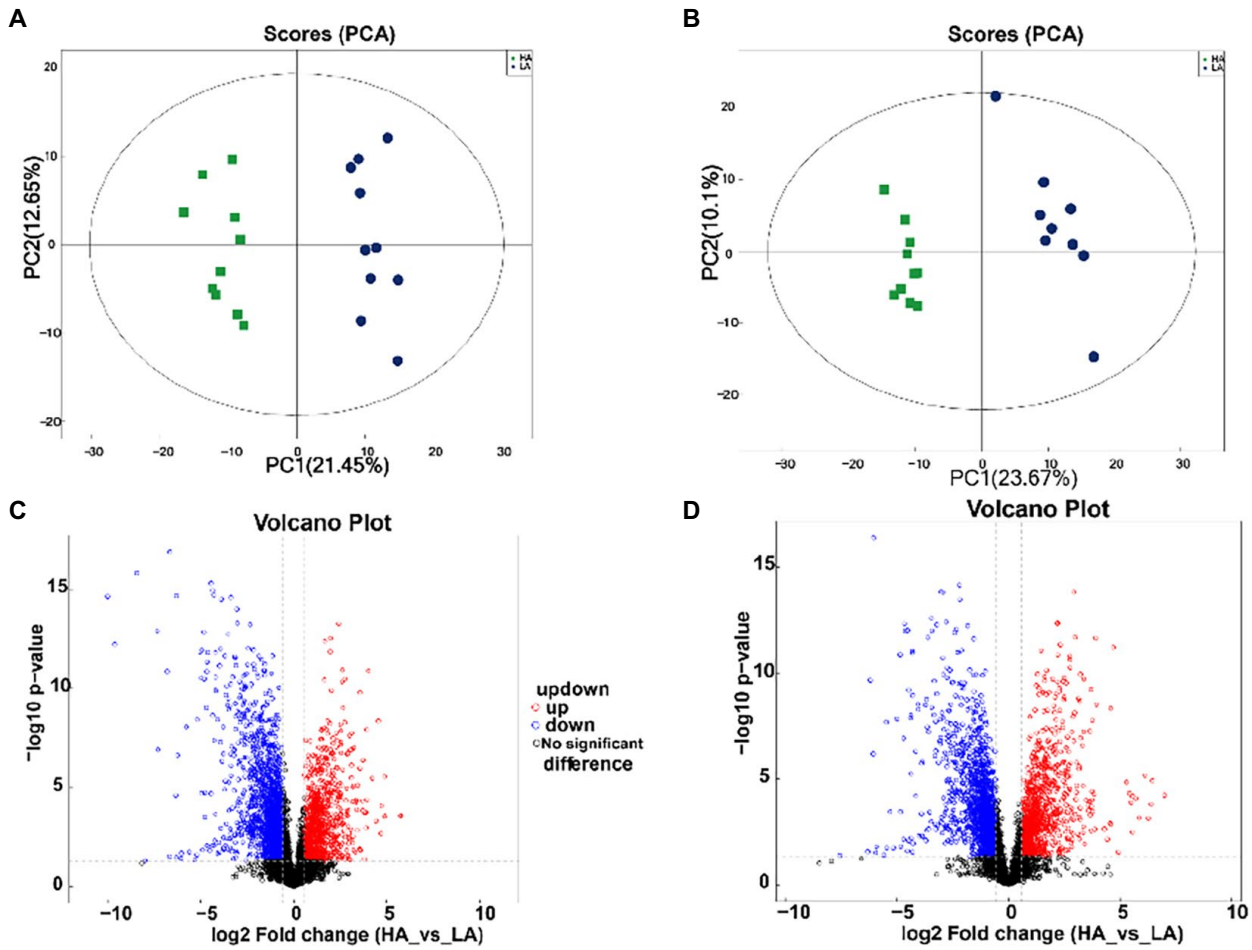
Correlation analysis of gut bacteria. **(A)** Heatmap of core operational taxonomic units (OTUs) (relative abundance >0.01% and *p* < 0.05 in all samples) significantly associated with metabolite production and fecal fermentative parameters in low-altitude (LA) and high-altitude (HA) Sanhe heifers, as determined by Spearman’s correlation analysis. *0.01 < *p* < 0.05, **0.001 < *p* < 0.01, ***p ≤ 0.001. **(B)** Interaction among OTUs with relative abundances > 0.01% and *p* < 0.05. The red and blue colors represent positive and negative correlations, respectively. The size and color of the circle represent the relative abundance of the OTUs and the genus to which they belong, respectively.

To explore the role of gut bacteria in production and fermentation of VFAs, we analyzed the relationship between fecal VFA concentration (acetate, propionate, butyrate, and total VFAs) and the relative abundance of OTUs using Spearman’s rank correlations, as shown in Figure 2. All OTUs with relative abundances <0.01% in all fecal samples were removed from the analysis. The relationship between OTUs and production and fermentation traits was visualized using a heat map (Figure 2). Fifty-Eight OTUs were significantly (*p* < 0.05) correlated with altitude; of these, 20 OTUs were negatively correlated with altitude, eight of which were in the family Ruminococcaceae (*p* < 0.05), four in the family Rikenellaceae (*p* < 0.05), and two in the family Lachnospiraceae (*p* < 0.05). Additionally, OTUs within unidentified_o_Clostridiales, Clostridiales_vadinBB60_group, Clostridiaceae_1, Bacteroidales_BS11_gut_group, Bacteroidales_RF16_group, and Spirochaetaceae were significantly negatively (*p* < 0.05) correlated with altitude. Thirty-eight OTUs were positively correlated with altitude, among which 14 were in the family Ruminococcaceae (*p* < 0.05), 11 in the family Christensenellaceae (*p* < 0.05), three in the family Family_XIII (*p* < 0.05), three in the family Lachnospiraceae (*p* < 0.05), and three in the family Peptostreptococcaceae (*p* < 0.05). In addition, OTUs within the families Erysipelotrichaceae, Acidaminococcaceae, Bacteroidaceae, and unidentified_o_Gastranaerophilales were significantly and positively correlated with altitude (*p* < 0.05).

Fourteen OTUs were negatively (*p* < 0.05) correlated with the acetate-to-propionate ratio (AP), among which six and three were in the families Ruminococcaceae and Rikenellaceae, respectively. In addition, 23 OTUs were positively correlated (*p* < 0.05) with AP, of which eight were in the family Ruminococcaceae, six in the family Christensenellaceae, three in the family Peptostreptococcaceae, and two in the family Lachnospiraceae. Furthermore, OTUs within the families Family_XIII, Erysipelotrichaceae, unidentified_o_Gastranaerophilales, and Acidaminococcaceae were significantly and positively correlated with AP (*p* < 0.05).

Analysis of VFAs showed that acetate concentration was negatively correlated with the relative abundance of OTUs in the *Eubacterium coprostanoligenes* group. Sixteen OTUs were significantly (*p* < 0.05) correlated with propionate concentration, among which 10 OTUs were negatively correlated with propionate concentration, three OTUs were in the family Christensenellaceae, three OTUs were in the family Peptostreptococcaceae, two OTUs were in the family Lachnospiraceae, and one OTU was in the family Erysipelotrichaceae. Six OTUs were significantly and positively (*p* < 0.05) correlated with propionate concentration; of these, four OTUs belonged to the family Ruminococcaceae, one OTU belonged to the family Bacteroidales_RF16_group, and one OTU belonged to the family Lachnospiraceae. The total VFA concentration was negatively (*p* < 0.05) correlated with the relative abundance within the family Ruminococcaceae.

### 3.3. Gut metabolome of Sanhe heifers from different altitudes

#### 3.3.1. Differential metabolites

**Figure 3:**
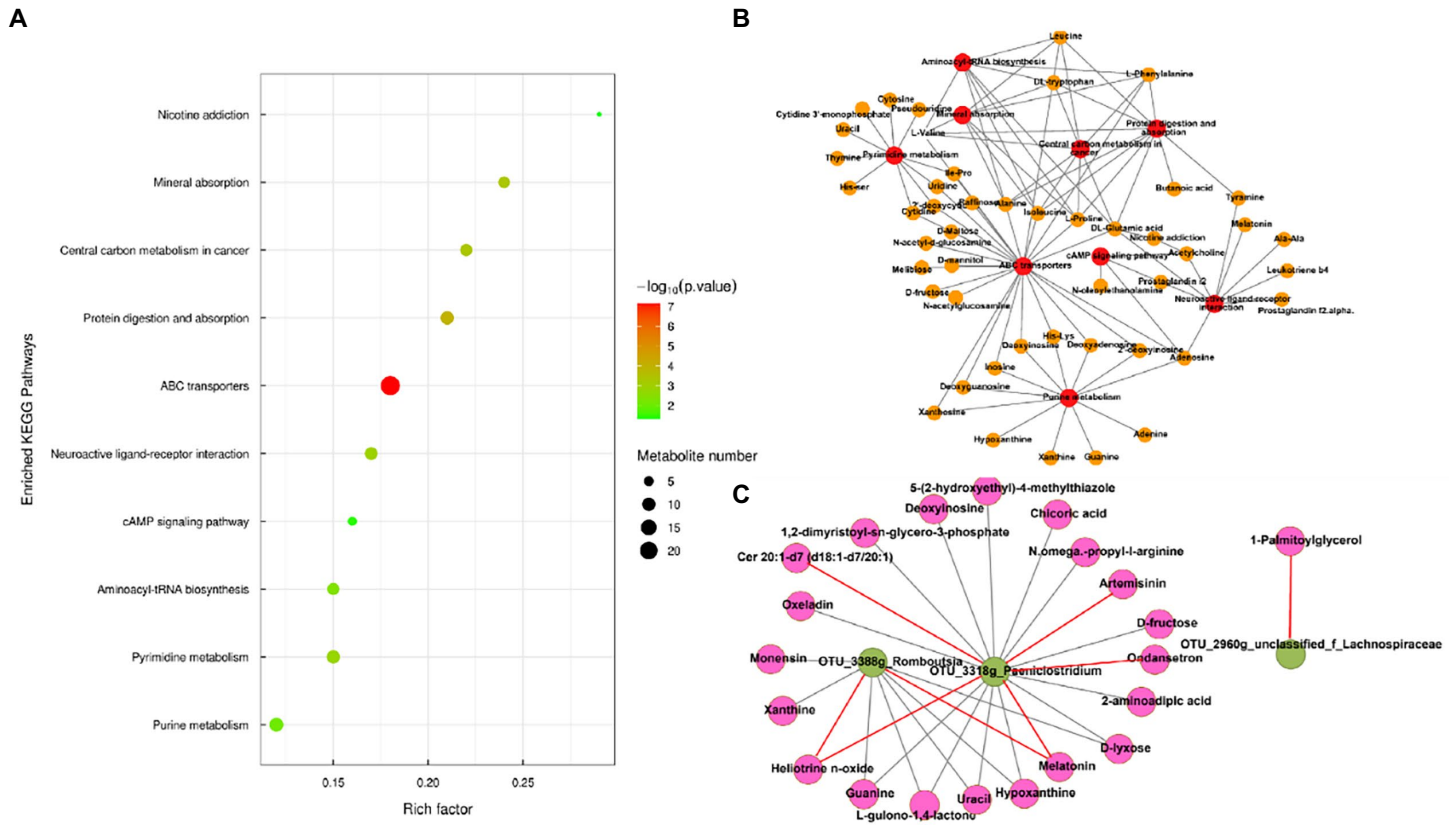
Metabolomic profiling of fecal samples. Scatter plots of the principal coordinate analysis model based on all identified metabolite features of gut samples from the two groups. [**(A)** positive mode; **(B)** negative mode]. The volcano plot of the comparison between the low-altitude (LA) and high-altitude (HA) groups [**(C)**, positive mode, **(D)**, negative mode].

A total of 1,727 differential metabolites were identified in the gut metabolome; of these, 1,101 and 626 metabolites were detected in positive and negative ion modes, respectively. To compare the metabolome compositions of the gut samples in the two groups, the datasets obtained from LC–MS in the positive and negative ion modes were evaluated using PCA (Figures 3A,B). The metabolites between the two groups were well-separated in the PCA score plots of the positive and negative ion mode results. Volcano plots of the positive and negative ion modes for the two groups are shown in Figures 3C,D. The OPLS-DA score plots are shown in Supplementary Figure S2. OPLS-DA revealed a clear distinction between the LA and HA groups in both the positive (R2X = 0.351, R2Ycum = 0.995, Q2cum = 0.954) and negative ion modes (R2X = 0.351, R2Ycum = 0.995, Q2cum = 0.954), which was validated by permutation analysis (positive: Q2 intercept = −0.2568; negative: Q2 intercept = −0.2413). Based on the cutoff (VIP >1 and *p* < 0.05) for differential metabolites, 368 metabolites differed significantly between the LA and HA groups, of which 231 and 137 were detected in the positive and negative ion modes, respectively.

#### 3.3.2. Kyoto encyclopedia of genes and genomes pathways

**Figure 4:**
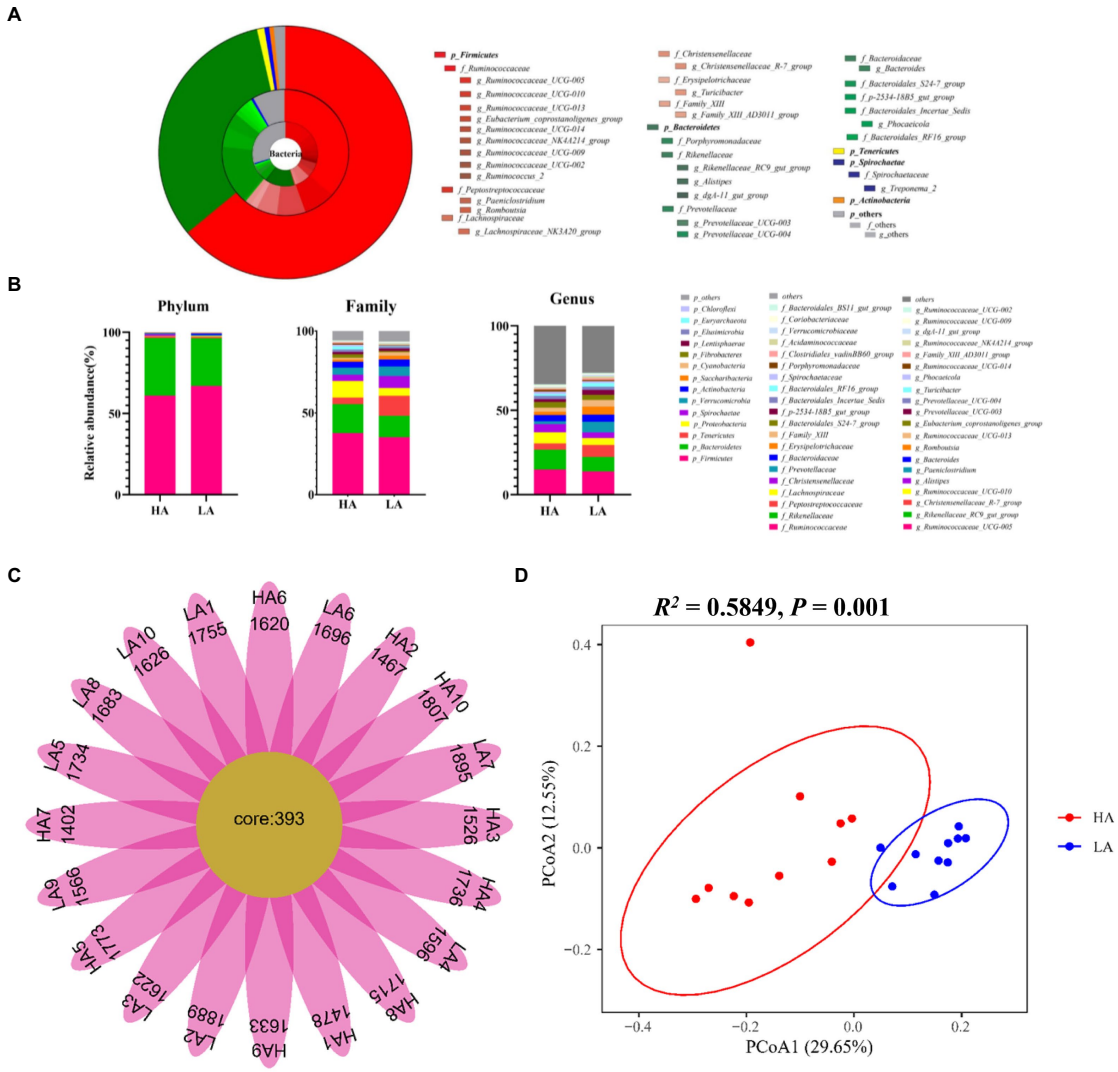
**(A)** Enriched Kyoto Encyclopedia of Genes and Genomes pathways of the comparison between the low-altitude (LA) and high-altitude (HA) groups (only those with a *p* < 0.05 are shown). **(B)** Network of enriched pathways and the differential metabolites between the LA and HA groups. **(C)** Correlation between gut core OTU (relative abundance > 0.1%) and differential metabolites in enrichment pathways based on **(A)** in the network (only those with |*r*| > 0.80, *p* < 0.05 are shown). Red and brown lines indicate positive and negative correlations, respectively.

Metabolic pathway analysis based on the significantly different gut metabolites revealed the enrichment of 10 metabolic pathways (Figure 4A), with “nicotine addiction,” “central carbon metabolism in cancer,” “mineral absorption,” “protein digestion and absorption,” “ABC transporters,” “neuroactive ligan-receptor interaction,” “cAMP signaling pathway,” “aminoacyl-tRNA biosynthesis,” “pyrimidine metabolism,” and “purine metabolism,” which belong to “environmental information processing,” “organismal systems,” “metabolism,” “human diseases,” and “genetic information processing,.” The differential metabolites in the differentially enriched KEGG pathways determined by hydrophilic interaction LC–MS analysis are shown in Table 4. In addition, the relationships between metabolic pathways were significantly different for the gut metabolites (Figure 4B).

**Table 4:** Differential metabolites in differential enriched KEGG pathways using HILIC-MS analysis.

HMDB^2^	Metabolites^1^	VIP	Fold-change	*P*-value	Direction^3^
HMDB0000012	Ile-Pro	7.242	9.081	<0.001	↑
HMDB0000085	Deoxyguanosine	2.842	4.355	<0.001	↑
HMDB0000296	Uridine	5.388	2.294	<0.001	↑
HMDB0000071	Deoxyinosine	3.348	5.345	<0.001	↑
HMDB0062538	D-fructose	2.622	3.145	<0.001	↑
HMDB0003213	Raffinose	1.577	3.502	<0.001	↑
HMDB0000883	L-Valine	1.163	1.955	<0.001	↑
HMDB0060475	DL-Glutamic acid	1.007	3.388	<0.001	↑
HMDB0000133	His-Lys	2.013	1.979	<0.001	↑
HMDB0033923	Isoleucine	1.720	1.706	0.002	↑
HMDB0000163	D-Maltose	1.638	2.122	0.002	↑
HMDB0000765	D-mannitol	1.232	0.083	0.002	↓
HMDB0000195	Inosine	1.959	1.613	0.005	↑
HMDB0000161	Alanine	1.280	4.901	<0.001	↑
HMDB0000101	Deoxyadenosine	13.257	4.300	<0.001	↑
HMDB0000162	L-Proline	1.002	2.457	<0.001	↑
HMDB0000299	Xanthosine	1.107	5.115	<0.001	↑
HMDB0000071	2′-deoxyinosine	1.744	5.884	<0.001	↑
HMDB0000048	Melibiose	3.664	2.290	<0.001	↑
HMDB0000050	Adenosine	8.671	3.117	0.002	↑
HMDB0000687	Leucine	2.191	2.023	0.003	↑
HMDB0000159	L-Phenylalanine	1.766	2.397	0.005	↑
HMDB0000014	2′-Deoxycytidine	3.750	5.029	0.006	↑
HMDB0000089	Cytidine	3.841	2.900	0.007	↑
HMDB0000215	N-acetyl-d-glucosamine	2.763	1.969	0.020	↑
HMDB0000215	N-Acetylglucosamine	1.524	4.979	0.023	↑
HMDB0001085	Leukotriene b4	1.131	1.785	<0.001	↑
HMDB0001335	Prostaglandin i2	7.465	1.610	0.002	↑
	Prostaglandin f2-alpha	1.235	0.608	0.003	↓
HMDB0001389	Melatonin	1.883	0.389	<0.001	↓
HMDB0000306	Tyramine	6.083	2.265	<0.001	↑
HMDB0000895	Acetylcholine	1.136	0.431	<0.001	↓
HMDB0000303	Ala-Ala	2.462	2.064	<0.001	↑
HMDB0002088	N-Oleoylethanolamine	1.582	1.387	0.007	↑
HMDB0030396	DL-Tryptophan	1.377	0.505	<0.001	↓
HMDB0000039	Butanoic acid	4.699	2.143	0.002	↑
HMDB0000300	Uracil	4.879	3.933	<0.001	↑
HMDB0000273	His-Ser	8.947	4.890	<0.001	↑
HMDB0000767	Pseudouridine	1.198	2.762	<0.001	↑
HMDB0000262	Thymine	5.749	6.776	<0.001	↑
HMDB0000630	Cytosine	2.951	2.281	<0.001	↑
	Cytidine 3′-monophosphate	4.811	2.991	<0.001	↑
HMDB0000292	Xanthine	4.591	6.458	<0.001	↑
HMDB0000132	Guanine	6.161	4.326	<0.001	↑
HMDB0000034	Adenine	7.059	4.201	<0.001	↑
HMDB0000157	Hypoxanthine	6.426	2.727	<0.001	↑

HILIC-MS, hydrophilic interaction liquid chromatography-mass spectrometry; VIP, variable important in projection. ^1^Metabolites were filtered using significance estimates of *p* < 0.05 and VIP > 1.0 (*n*¼5). ^2^HMDB of metabolites in human metabolome database. ^3^‘↑’ indicates the metabolite in the HA group was increased when compared to those in the LA group. ‘↓’ indicates the metabolite in the HA group was decreased when compared to those in the LA group.

### 3.4. Relationships between the core gut microbiota and metabolites

Spearman’s correlation network between the core gut microbiota and gut metabolites was analyzed, that revealed 28 significant correlations (relative abundance >0.1%, *r* > |0.8|, *p* < 0.05; Figure 4C). OTUs belonging to the genus *Romboutsia* were significantly negatively and positively correlated with seven and two metabolites. Respectively. OTUs belonging to the genus *Paeniclostridium* were significantly negatively and positively correlated with 13 and five metabolites, respectively. OTUs belonging to the genus *unclassified_f_Lachnospiraceae* were significantly positively correlated with a single metabolite.

## 4. Discussion

By integrating gut 16S rRNA high-throughput sequencing and LC–MS-based untargeted metabolomic analyzes, we investigated the gut microbiome and host metabolome mechanisms involved in high-altitude adaptability. We estimated the gut microbial composition, metabolites, and variations as well as the interactions between microorganisms and metabolites in different groups.

### 4.1. Gut microbiota communities of Sanhe heifers reared in different altitudes

VFAs did not differ in ruminal samples from cattle reared in different regions, suggesting that altitude does not strongly affect the fecal fermentation parameters in Sanhe heifers.

Generally, the intestinal microbiota is stable over time in adult animals (Caporaso et al., 2011; Faith et al., 2013). In this study, we investigated the differences in the gut microbiota of Sanhe heifers reared at different altitudes. In the two groups, Firmicutes and Bacteroidetes, known to play a key role in maintaining gut homeostasis, were the most abundant phyla in the gut of Sanhe heifers, which agrees with the findings observed in yaks (Liu et al., 2021) and rats. Members of Bacteroides participate in the degradation of biopolymers and main polysaccharides, whereas bacteria from Firmicutes regulate the digestion and absorption of proteins and carbohydrates. At the phylum level, the enrichment of Proteobacteria in the gut represents an imbalanced and unstable microbiota structure or disease state in the host (Shin et al., 2015). Actinobacteria are thought to be involved in modulating gut permeability, immune system, metabolism, and the gut-brain axis, and their abundance represents the health state of the animal. The relative abundance of Spirochaetae was lower in the gut of HA heifers than in that of LA heifers, as observed previously in sub-adult Tibetan sheep (Li et al., 2020), which are saccharolytic and can use carbohydrates as substrates. Families showing differential abundances, including Peptostreptococcaceae, Christensenellaceae (Waters and Ley, 2019), Erysipelotrichaceae (Wu et al., 2021), Family_XIII, and Lachnospiraceae, *Domibacillus* (Sharma et al., 2014), Bacteroidales_S24-7_group (Gao et al., 2020), Bacteroidales_RF16_group, and Porphyromonadaceae (Sakamoto, 2014), most of which belonged to the phyla Firmicutes, Bacteroidetes, Actinobacteria, and Spirochaetae, are associated with fiber degradation, feed digestion, and inflammation induction. *Butyrivibrio* (Kelly et al., 2010) and *Eubacterium_xylanophilum_group* (Mukherjee et al., 2020) are butyrate-forming bacteria that play key roles in polysaccharide degradation. The relative abundances of *Eubacterium_xylanophilum_group* (Mukherjee et al., 2020), *Corynebacterium* (Salem et al., 2015), *Escherichia-Shigella* (The et al., 2016), and *Domibacillus* vary widely, and most of these organisms are pathogens, suggesting that changes in altitude affect the structure of the intestinal microbiota and the health of Sanhe heifers. Moreover, we considered the impact of altitude on the gut bacterial core OTUs of Sanhe heifers. Therefore, these OTUs may form the key bacterial community responsible for high-altitude adaptibilty in Sanhe heifers.

### 4.2. Gut metabolome of Sanhe heifers in different altitudes

The enriched differential metabolic pathways belonged to nucleotide metabolism, including pyrimidine and purine metabolism pathways. Purine and pyrimidine nucleotides are major energy carriers, subunits of nucleic acids, and precursors for the synthesis of nucleotide cofactors (Moffatt and Ashihara, 2002). The enriched differential metabolic pathways belonged to the digestive system of organismal systems, including mineral and protein digestion and absorption, suggesting that different altitudes affect the digestive system of Sanhe heifers. The enriched differential metabolic pathways belonged to environmental information processing, including “ABC transporters,” “neuroactive ligan-receptor interaction,” and “cAMP signaling pathway.” The cAMP signaling pathway regulates critical physiological processes, including metabolism, secretion, calcium homeostasis, muscle contraction, cell fate, and gene transcription (Ould Amer and Hebert-Chatelain, 2018). The cyclic nucleotide-gated ion channel regulates downstream pathways by activating calmodulin and calcium/calmodulin-dependent protein kinase. In addition, the cAMP pathway, also known as the protein kinase A pathway, directly regulates the transmembrane transport of calcium, potassium, sodium, and chloride ions through phosphorylation of channel proteins, transporters, and receptors on the cell membrane. ABC transporters exert a variety of physiological functions, such as the removal of foreign substances, nutrient intake, resistance to foreign invasion, antigen transmission, and inhibition of transportation, and are closely related to the health of the body (Liu, 2019; Thomas and Tampé, 2020). All these pathways were upregulated in the HA group compared to those in the LA group. In addition, the KEGG pathway was enriched in human diseases, suggesting that high altitudes affect the health of Sanhe heifers. Overall, untargeted metabolomics showed that high-altitude regions could alter organismal systems, metabolism, environmental information processing, genetic information processing, and even induce disease. Altitude also affects environmental information processing, organismal systems, human diseases, and genetic information processing.

### 4.3. Relationships between the core gut microbiome and gut metabolites

We found that OTUs belonging to the genus *Romboutsia* were associated with nine metabolites (melatonin, uracil, hypoxanthine, xanthine, guanine, monensin, heliotrine N-oxide, d-lyxose, and L-gulono-1,4-lactone). A previous study showed that *Romboutsia* encodes a versatile array of metabolic capabilities involved in carbohydrate utilization, fermentation of single amino acids, anaerobic respiration, and metabolic end**-**products (Gerrritsen et al., 2019), which is consistent with our results. OTUs belonging to the genus *Paeniclostridium* were associated with 18 metabolites (L-gulono-1,4-lactone, uracil, d-lyxose, chicoric acid, guanine, 5-(2-hydroxyethyl)-4-methyl thiazole, oxeladin, hypoxanthine, 2-aminoadipic acid, melatonin, 1,2-dimyristoyl-*sn*-glycero-3-phosphate, N-omega-propyl-l-arginine, artemisinin, heliotrine N-oxide, ondansetron, Cer 20:1-d7 (d18:1-d7/20:1), deoxyinosine, and d-fructose). *Paeniclostridium* is an anaerobic pathogen in animals (Kim et al., 2017). The OTUs belonged to the genus *g_unclassified_f_Lachnospiraceae* which is a member of the family Lachnospiraceae that is positively correlated with 1-palmitoylglycerol. Previous studies have shown that insoluble fatty acid soap might reduce the growth benefits in the intestine (Yaron et al., 2013; Wang et al., 2020), suggesting that excess 1-palmitoylglycerol causes intestinal damage. Therefore, Sanhe heifers are more prone to diseases in high-altitude environments.

Overall, our results showed that the gut microbiome and metabolome of Sanhe heifers differed between the LA and HA groups. We found that the gut microbiota associated with digestion absorption of proteins and carbohydrates, including Peptostreptococcaceae, Christensenellaceae, Erysipelotrichaceae, Family_XIII, Lachnospiraceae, *Domibacillus*, Bacteroidales_S24-7_group, Bacteroidales_RF16_group, Porphyromonadaceae, and Spirochaetaceae, differed between HA heifers and LA heifers. These findings indicate that the ability of the gut microbiota to ferment dietary substrates differs between LA and HA Sanhe heifers. The core OTUs in the phyla Bacteroidetes, Firmicutes, Spirochaetes, and Cyanobacteria differed between the gut microbiota of the LA and HA groups. Therefore, these organisms may be critical bacterial communities involved in determining the high-altitude adaptabilty of Sanhe heifers. In addition, untargeted metabolomics has shown that high-altitude regions could alter organismal systems, metabolism, environmental information processing, genetic information processing, and even induce diseases. The genera *Romboutsia*, *Paeniclostridium*, and *g_unclassified_f_Lachnospiraceae* were strongly associated with the 28 differential metabolites. In summary, when Sanhe heifers encounter the stress of high-altitude environments, they respond by regulating their gut microbiome and metabolome; however, changes in altitude negatively affect the digestive ability and health of Sanhe heifers. This study contributes to the understanding of the ability of dairy cows to adapt to high-altitude regions and provides insights into strategies for altering the gut microbiota for high-altitude adaptation through feeding management.

## 5. Conclusion

We investigated the gut microbiome and metabolome mechanisms involved in the adaptation to high-altitude environments. Variations in the gut microbiome and metabolome, as well as the interaction of microorganisms and metabolites, were studied in the LA and HA groups by integrating gut 16S rRNA high-throughput sequencing and LC–MS-based untargeted metabolomic analyzes.

## Data availability statement

The datasets presented in this study can be found in online repositories. The names of the repository/repositories and accession number(s) can be found in the article/Supplementary material. The sequence data supporting the results of this study are available in the NCBI Sequence Read Archive (SRA) under the accession number PRJNA821486.

## Ethics statement

The animal study was reviewed and approved by the study protocol was approved by the Ethical Committee of the College of Animal Science and Technology of China Agricultural University (project number AW22121202-1-2). Written informed consent was obtained from the owners for the participation of their animals in this study.

## Author contributions

XZ performed experiments and wrote the manuscript. WW, ZC, HY, YW, and SL reviewed and provided guidance for the manuscript and experiment. All authors contributed to the article and approved the submitted version.

## Funding

The services used in this study were purchased by the Ministry of Agriculture and Rural Affairs of China: Experiment and Demonstration of Adaptive Production Technology for Dairy Cows in High Altitude Regions (no.16190319) and China Agriculture Research System of MOF and MARA (CARS36).

## Conflict of interest

The authors declare that the research was conducted in the absence of any commercial or financial relationships that could be construed as a potential conflict of interest.

## Publisher’s note

All claims expressed in this article are solely those of the authors and do not necessarily represent those of their affiliated organizations, or those of the publisher, the editors and the reviewers. Any product that may be evaluated in this article, or claim that may be made by its manufacturer, is not guaranteed or endorsed by the publisher.

## Footnotes

^1^ www.metaboanalyst.ca

^2^ http://www.kegg.jp/
