# Supplementary material for: Effects of altitude on the gut microbiome and metabolomics of Sanhe heifers
Source: Front Microbiol. 2023 Feb 24;14:1076011. doi: 10.3389/fmicb.2023.1076011 (PMC10002979; doi:10.3389/fmicb.2023.1076011)
Supplement: Supplementary file 1 [file Data_Sheet_1.docx]

**Table S1. The nutrient levels of the TMR (DM, %).**

| Items  Items | LA  Control | HA  HA |
| --- | --- | --- |
| Wheat bran | 12.07 | --- |
| Highland barley bran | --- | 10.59 |
| Corn silage | 29.69 | 29.59 |
| Wheat straw | 10.94 | --- |
| Leymus chinensis hay | 7.81 | --- |
| Oat hay | --- | 18.65 |
| Alfafa hay | --- | 7.84 |
| Whole cottonseed | 3.12 | 2.98 |
| Steam-flaked corn | --- | 15.14 |
| Cottonseed meal | 4.79 | 6.40 |
| Soybean meal | --- | 5.86 |
| Barley | 15.03 | --- |
| Rapeseed meal | 13.60 | --- |
| Sodium bicarbonate | 0.20 | 0.20 |
| Slow-release urea supplement | 0.30 | 0.30 |
| Dicalcium phosphate | 0.30 | 0.30 |
| Limestone | 0.60 | 0.60 |
| Salt | 0.30 | 0.30 |
| Premix^1^ | 1.25 | 1.25 |
| Total | 100.00 | 100.00 |
| Nutritional levels^2^ | | |
| Dry matter (DM) | 50.79 | 51.30 |
| Organic matter (OM) | 90.45 | 91.05 |
| Crude protein (CP) | 15.70 | 15.78 |
| Ether extract (EE) | 2.92 | 2.91 |
| Neutral detergent (NDF) fiber (NDF) | 38.98 | 38.85 |
| Acid detergent fiber (ADF) | 22.36 | 22.79 |
| Ca | 0.78 | 0.83 |
| P | 0.44 | 0.46 |
| NE_M_,^3^ MJ /kg | 6.70 | 6.82 |
| NE_G_,^4^ MJ/kg | 4.61 | 4.74 |

Note:^1^Premix provided the following per kg for the diets of Sanhe heifers: vitamin A, 265,000IU; vitamin D_3_, 110,200 IU; vitamin E, 23,000 IU; Mn, 0.25%; Fe, 0.25%; Zn, 0.25%; Cu, 15 mg; I, 265,000 IU; Se, 30 mg.

^2^Nutrient composition: They were actual measured values.

^3^NE_M_, [net energy for maintainance](http://m.shortof.com/suolueci/nem-net-energy-maintainance)

^4^NE_G_, net energy for growth

**Table S2 Effects of different altitude regions on α-diversity of feces microbiota of the Sanhe heifers**

| Items | Groups^1^ | | SEM | *P-*value |
| --- | --- | --- | --- | --- |
|  | LA | HA |  |  |
| OTUs | 2008.70 | 2099.20 | 29.91 | 0.13 |
| goods_coverage | 0.97 | 0.97 | 0.001 | 0.18 |
| observed_species | 2098.92 | 2008.54 | 29.90 | 0.13 |
| Chao1 | 2734.03 | 2615.36 | 35.20 | 0.09 |
| Shannon | 8.46 | 8.38 | 0.11 | 0.73 |
| Simpson | 0.99 | 0.98 | 0.005 | 0.37 |

^1^LA represents the low-altitude region (Hulunbuir City, Inner Mongolia Autonomous Region, 119°57 'E, 47°17' N; about 700 m altitudes, LA); HA represents the high-altitude region (Lhasa City, Tibet Autonomous Region 91°06'E, 29°36'N; about 3 750 m altitudes, HA).

# Figure S1 | Individual OTU rarefaction curves for each Sanhe heifer feces sample take.


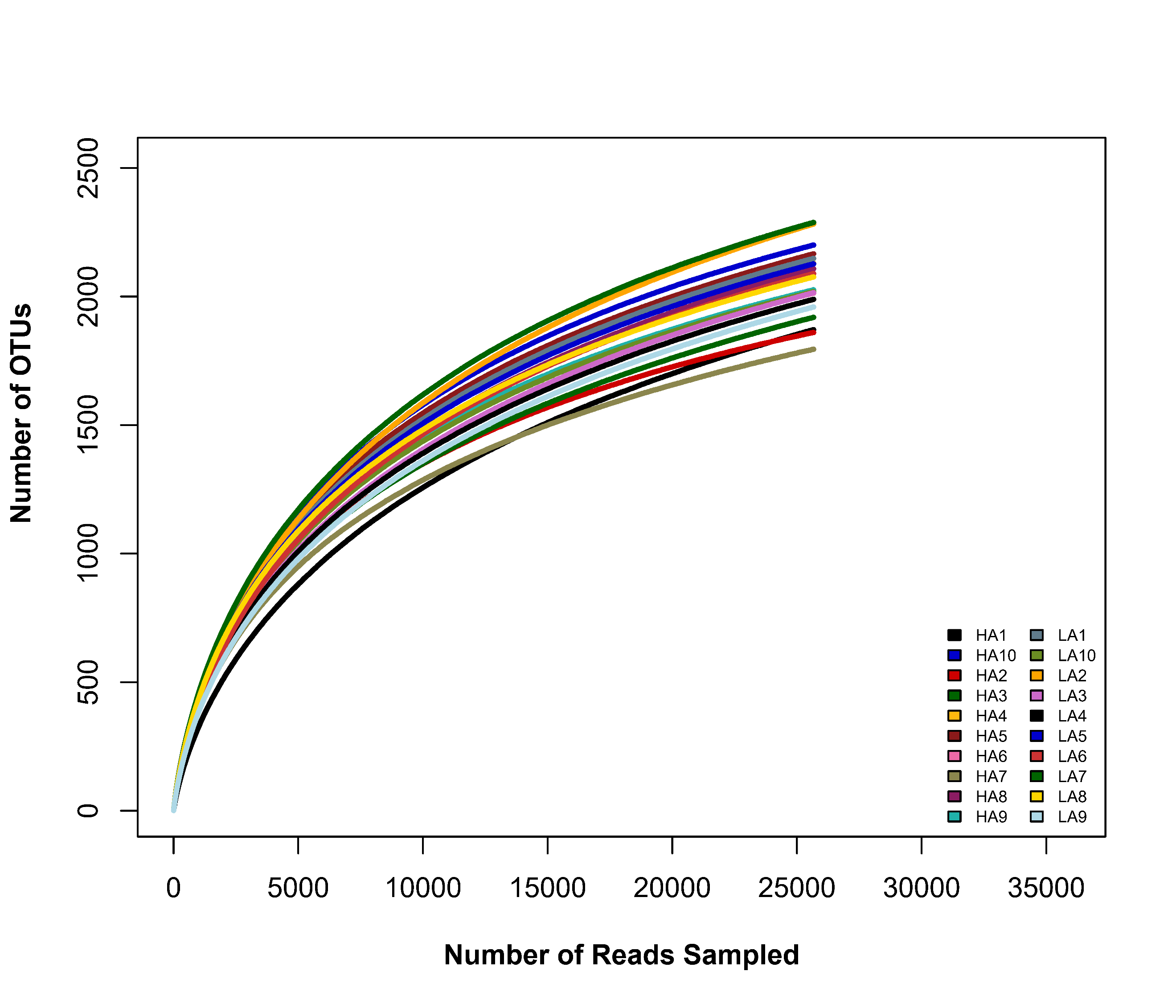


# Figure S2 | OPLS-DA scores plots for the feces samples obtained from the HILIC-MS analysis between LA and HA Sanhe heifers.


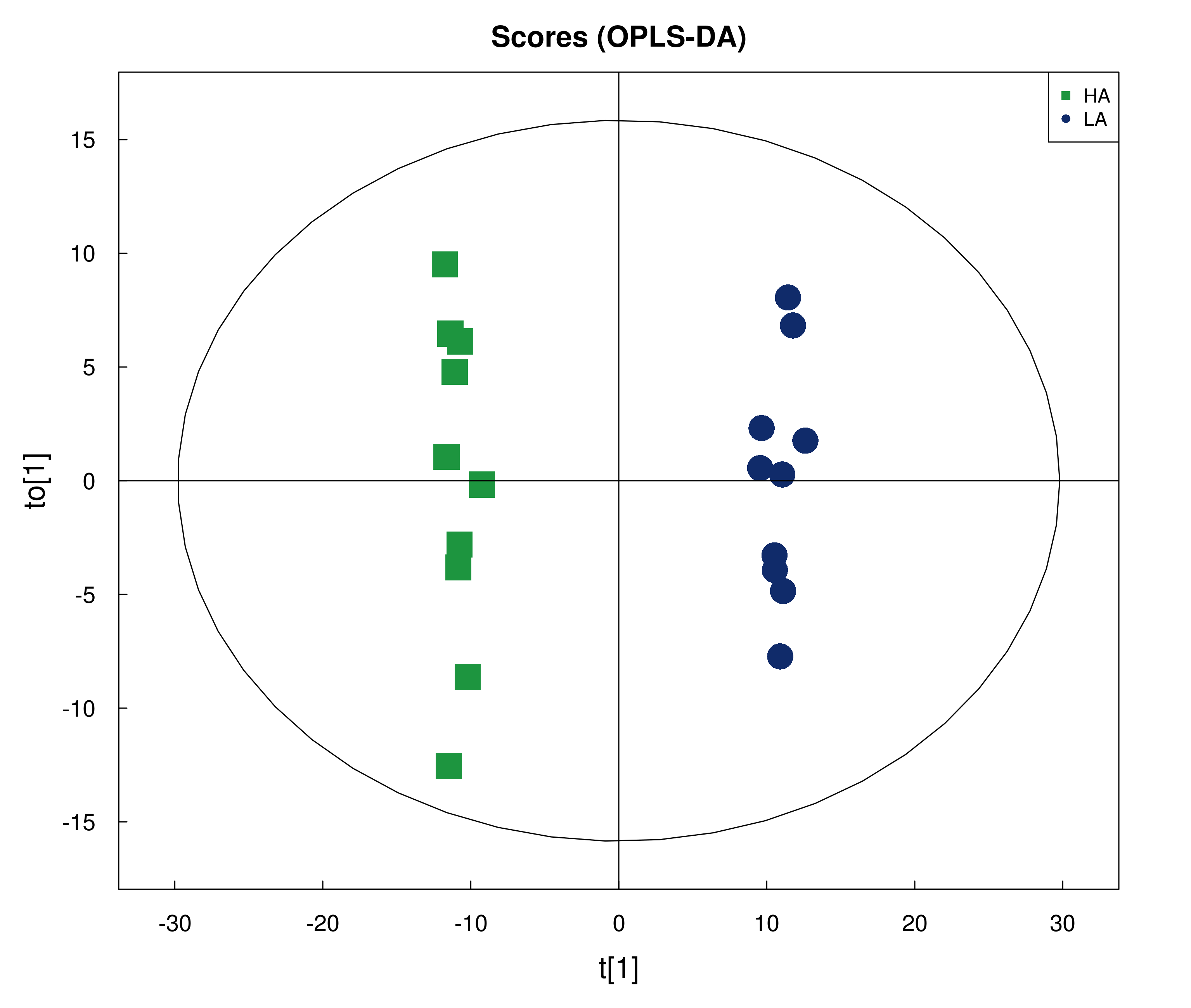

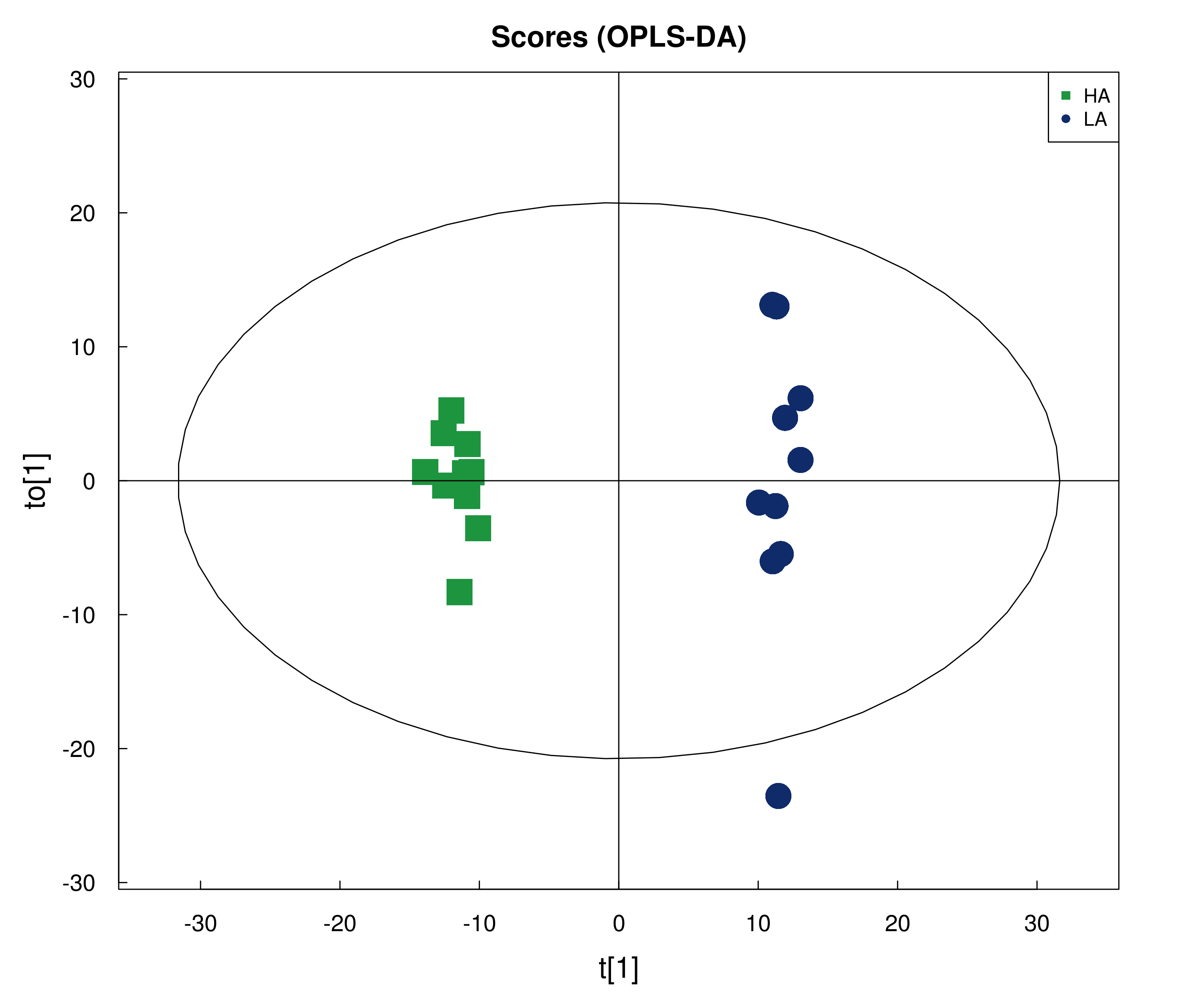


# LA represents the low-altitude region (Hulunbuir City, Inner Mongolia Autonomous Region, 119°57 'E, 47°17' N; about 700 m altitudes, LA); HA represents the high-altitude region (Lhasa City, Tibet Autonomous Region 91°06'E, 29°36'N; about 3 750 m altitudes, HA). The X-axis indicated score of the principal component. The Y-axis indicated orthogonal T score. OPLS-DA¼orthogonal partial least squares discriminant analysis; HILIC-MS¼hydrophilic interaction liquid chromatography-mass spectrometry. Violin plots of number of OTUs, Chao1 richness and Shannon diversity index between LA and HA.
